# Selection and validation of reference genes desirable for gene expression analysis by qRT-PCR in MeJA-treated ginseng hairy roots

**DOI:** 10.1371/journal.pone.0226168

**Published:** 2019-12-05

**Authors:** Li Li, Kangyu Wang, Mingzhu Zhao, Shaokun Li, Yue Jiang, Lei Zhu, Jing Chen, Yanfang Wang, Chunyu Sun, Ping Chen, Jun Lei, Meiping Zhang, Yi Wang

**Affiliations:** 1 College of Life Science, Jilin Agricultural University, Changchun, Jilin, China; 2 Research Center of Ginseng Genetic Resources Development and Utilization, Changchun, Jilin, China; 3 College of Chinese Medicinal Materials, Jilin Agricultural University, Changchun, Jilin, China; 4 Key Laboratory of Natural Resources of Changbai Mountain & Functional Molecules (Yanbian University), Ministry of Education, Yanji, Jilin, China; Macau University of Science and Technology, MACAO

## Abstract

Ginseng is a valuable herb of traditional Chinese medicine and ginsenosides, the main bioactive components of ginseng, have been proven to have multiple functions in human therapies and health. Methyl jasmonate (MeJA) is an elicitor that has been demonstrated to have a vital influence on ginsenoside biosynthesis. Quantitative real-time polymerase chain reaction (qRT-PCR) has been widely used in quantification of gene expressions. Here, we report the selection and validation of reference genes desirable for normalization of gene expressions quantified by qRT-PCR in ginseng hairy roots treated with MeJA. Twelve reference genes were selected as candidate genes, and their expressions were quantified by qRT-PCR, and analyzed by geNorm, NormFinder and BestKeeper. *CYP* and *EF-1α* were shown to be the most stable reference genes in geNorm, *CYP* was the most stable reference gene in NormFinder, and *18S* was the most stable reference gene in BestKeeper. On this basis, we further quantified the relative expression levels of four genes encoding key enzymes that are involved in ginsenoside biosynthesis using *CYP* and *18S* as the reference genes, respectively. Moreover, correlation analysis was performed between the quantified expressions of four genes and the ginsenoside content in MeJA-treated ginseng hairy roots. The results of relative expressions of the four genes quantified using *CYP* as the reference gene and their significant correlations with the ginsenoside content were better than those using *18S* as the reference gene. The *CYP* gene, hence, was concluded as the most desirable reference gene for quantification of the expressions of genes in MeJA-treated ginseng hairy roots. This finding, therefore, provides information useful for gene research in ginseng, particularly in MeJA-treated ginseng hairy roots, which includes identification and characterization of genes involved in ginsenoside biosynthesis.

## 1. Introduction

Ginseng (*Panax ginseng* C.A. Meyer) is a valuable herb of traditional Chinese medicine that has curative effects on several human diseases, including cancerous[1], inflammatory[2], cardiovascular[3] and neurodegenerative diseases[4]. It also has effects on reducing obesity and the regulation of immunity[5]. Nevertheless, ginseng research is facing several challenges. For instance, ginseng must grow at least four years before it could be effectively used in medicine, during which it is likely subjected to a variety of diseases. Wild ginseng is rare and thus, is extremely valuable. Therefore, several tissue culture methods, such as callus, somatic embryogenesis, adventitious roots and hairy roots culture[6] have been developed and used to generate plant materials for ginseng research. Among the plant materials generated through these methods, hairy roots are desirable for numerous aspects of ginseng research since they contain components that are very similar to native ginseng, grow rapidly and are hormone-autotrophic. Ginsenosides are the main bioactive components in ginseng, including protopanaxadiol (PPD)-type, protopanaxatriol (PPT)-type, oleanolic acid-type and ocotillo-type[7]. As the secondary metabolites, ginsenosides also play a vital role in plant defense to biotic and abiotic stresses. More than 150 naturally occurring ginsenosides have been so far identified from *Panax* species[8], but their types and content vary greatly among tissues and through periods of growth and development. It was reported that the ginsenoside content was the highest in leaves and the lowest in primary roots. The content ratio of PPT-type and PPD-type ginsenoside was about 1.0 in primary roots, while it was 1.37–3.14 in leaves[9]. Moreover, the tissue-specific content and distributions of ginsenosides could be changed by elicitors through controlling growth conditions, for which the elicitors used for such purposes including fungi[10], N, N-dicyclohexylcarbodiimide (DCCD)[11], salicylic acid (SA)[12] and Methyl jasmonate (MeJA)[13]. Among these elicitors, MeJA has been proven to be the most effective and therefore, been used most widely. It has been reported that MeJA has a vital influence on the expression of the genes involved in ginsenoside biosynthesis and ginsenoside content in hairy roots[14, 15], adventitious roots[16, 17] and other tissues[18]. Therefore, these elicitors can be potentially used for increasing ginsenoside production, identifying genes involved in ginsenoside biosynthesis, as well as studying the molecular mechanisms of ginsenoside biosynthesis and regulation. Especially, the MeJA-treated ginseng hairy roots have been widely used in the study of ginsenoside biosynthesis.

Quantitative real-time polymerase chain reaction (qRT-PCR) has been widely used for quantification of gene expressions[19]. The reference gene used for gene expression quantification by qRT-PCR is a vital factor to normalize gene differential expression among samples, but its expression often substantially varies with tissues, treatments and growth conditions[20]. Liu et al.[21] investigated 20 genes as the reference genes for qRT-PCR at different growth stages and in different tissues for ginseng research. Of these, 20 candidate genes studied, 10 were traditional housekeeping genes and 10 were novel genes selected by transcriptome analysis. Later, Wang et al.[22] also conducted a similar study in different tissues of ginseng and ginseng seedlings treated with heat stress. Nevertheless, reference genes that were desirable for ginseng research using hairy roots grown under the stress or elicited condition, especially those treated with MeJA, have not been reported.

Several sorts of software have been used to test the stability of reference genes for gene expression analysis by qRT-PCR, including geNorm, NormFinder, RefFinder and Bestkeeper. They all calculate the stability of reference genes based on the Ct value. Recently, a validation step has been included in the process, following the Ct value calculation[23–28]. Unfortunately, only genes that are sensitive to treatment, such as *HSF* (heat shock protein), were used for reference gene validation, and the reference gene was believed to be suitable when its expression level significantly changed in different conditions. However, it is essential and desirable to further validate the reference gene by examining the expressions of a selection of targeted genes and the correlation of their expression with traits.

In the present study, we treated the ginseng hairy roots with MeJA, determined the change of its ginsenoside content, selected the reference genes for gene expression analysis by qRT-PCR, and validated the desirable reference gene by correlation analysis between ginsenoside content and the expressions of genes involved in ginsenosides biosynthesis. The reference genes selected and validated in this study, therefore, will facilitate the identification and characterization of genes involved in ginsenoside biosynthesis.

## 2. Materials and methods

### 2.1 Plant material and MeJA treatment

Ginseng cultivar ‘Damaya’ was used as plant materials for hairy root inducing in this study. After washed and sterilized, the ginseng seeds were germinated on 1/2 MS medium and cultured at 23°C for 15 days till the seedlings leaf fully unfolded. The seedlings were cut into 0.5 cm segments and dipped into *Agrobacterium rhizogenes* strain A4 for 15 min. After infection, the explants were dried on sterilized filter papers, and cultured at 23°C in dark on 1/2 MS medium containing 200 mg/L acetosyringone. After 2 days of co-cultivation, the explants were transferred to 1/2 MS medium containing 50 mg/L kanamycin and 500 mg/L cefotaxime to eliminate the *Agrobacterium*. Putative hairy roots were obtained approximately in a month after selection (50 mg/L kanamycin). Every induced single hairy root was individually further cultured to ensure the homogeneity of genetic background. The fastest-growing hairy root line was used as the materials for MeJA treatment. The ginseng hairy roots for MeJA treatment were cultured in flasks containing 150 ml 1/2 MS liquid medium with an initial inoculation of 1.0 g hairy roots at 22 ^o^C with shaking at 110 rpm. After cultured for 23 days, the hairy roots were treated with 200 μM MeJA that was previously shown to be the most desirable for the MeJA treatment in our laboratory. The hairy roots were harvested after treatment at 6 h, 12 h, 24 h, 48 h, 72 h, 96 h and 120 h, respectively. Three biologically duplicated samples and one blank control with no MeJA treatment were harvested at every time point. A part of hairy root samples was flash-frozen in liquid nitrogen for RNA extraction and the remaining part was dried to constant weight for ginsenoside extraction.

### 2.2 Ginsenoside extraction and content quantification

Ginsenosides were extracted by the Soxhlet extraction method[29]. Weighed samples (0.5 g) were wrapped up with filter paper and transferred into a 250 ml refluxing-type Soxhlet extractor. The samples were extracted by 100 ml methanol in 90 ^o^C water bath for 12 h, with every sample having three technical replicates. The extracts were collected and steamed to dry, and then the residue was dissolved by 10 ml chromatographic methanol, and filtered for analysis.

The mono-ginsenosides in each sample were separated using the Waters Alliance HPLC, with e2695 Separations Module, and their content was determined using the Waters 2489 Ultraviolet Spectrophotometric Detector (Waters, Milford, MA, USA). A Waters Uxbridge C18 column (4.6 mm × 250 mm, 5 μm) was used to achieve the separation. The mobile phase containing Solvent A (acetonitrile) and Solvent B (water) was set to a gradient elution program as reported by Cong et al.[30]: 0–40 min (18–21% A), 40–42 min (21–26% A), 42–46 min (26–32% A), 46–66 min (32–33.5% A), 66–71 min (33.5–38% A), 71–86 min (38–65% A), 86–91 min (65% A), 91–96 min (65–85% A), 96–103 min (85% A), 103–105 min (85–18% A) and 105–106 min (18% A). The flow rate was 1.0 ml/min, the sample injection volume was 20 μL, the column oven temperature was 35 ^o^C, and the detection wavelength was 203 nm. The content of total ginsenoside was examined by the Vanillin colorimetric method as reported[31].

### 2.3 Total RNA isolation and cDNA synthesis

Total RNA of ginseng hairy roots was isolated using the TRIpure Reagent Total RNA Extraction Reagent (Bioteke, Beijing, China). The concentration and quality of the RNA were determined by Scandrop 100 (Analytic Jena AG). cDNA synthesis from the RNA was carried out using the PrimeScript^™^ RT Reagent Kit with gDNA Eraser (Perfect Real Time) (TaKaRa, Dalian, China). The concentration of cDNA was also determined by Scandrop 100 and then diluted to 100 ng/μL for qRT-PCR.

### 2.4 Candidate reference genes and key enzyme coding genes of ginsenoside biosynthesis for qRT-PCR

Based on the previous studies in ginseng[21, 22], 12 widely used qRT-PCR reference genes were selected as candidate genes, including *18S* (*18S ribosomal RNA*), *ACT1* (*actin 1*), *aTUB* (*tubulin alpha-1 chain*), *bTUB* (*beta-tubulin*), *CYC* (*cyclophilin ABH-like protein*), *CYP* (*cyclophilin*), *EF-1α* (*elongation factor 1-alpha*), *eIF-5A* (*translational initiation factor eIF-5a*), *F-box* (*F-box containing protein*), *GAPDH* (*glyceraldehyde-3-phosphate dehydrogenase*), *IF3G1* (*eukaryotic translation initiation factor 3G1*), and *UBQ* (*polyubiquitin*). The primers of these genes reported by these researchers were used in this study. Four already verified key enzyme genes in mevalonate pathway of ginsenoside biosynthesis were randomly selected as the positive controls for validation of the reference genes by qRT-PCR, including *PgFPS*[32], (DQ087959.1, F-GGATGATTATCTGGATTGCTTTGG, R-CAGTGCTTTTACTACCAACCAGGAG) *PgDDS*[33] (AB122080.1, F- CGGAACGATTGACACTATTCTGAC, R- CTGACCCAATCATCGTGCTGT), *PgUGT71A27* (*UGTPg1*)[34] (KM491309.1, F- TGCGTCCGTCTATCCCTAAAG, R- TGATGTCCTGTCCAAGAATCCTAC) and *PgCYP716A47* [35] (JN604536.1, F- TTAGGTGATACGGCGGCAG, R-CTGGGGGATGCGTTTTGTAT).

### 2.5 qRT-PCR

qRT-PCR was carried out with the ABI 7500 Real-Time PCR System (Applied Biosystem, Foster City, CA, USA) and TB Green^™^ Premix Ex Taq^™^ (Tli RNase H Plus) (TaKaRa, Dalian, China). The reaction system included 10 *μ*L TB Green^™^ Premix Ex Taq^™^ (2X), 0.4 *μ*L Rox Ⅱ, 0.8 *μ*L each primer, 1 *μ*L cDNA and 7 *μ*L RNase-free water. The reaction was performed as follows: pre-denaturation at 95 ^o^C for 30 s; PCR of 40 cycles at 95 ^o^C for 5 s and 60 ^o^C for 34 s; melting curve at 95 ^o^C for 15 s, 60 ^o^C for 1 min, and 95 ^o^C for 15 s. Each biological sample was tested in triplicate. The gene relative expression levels were calculated by the 2^-ΔΔCt^ method.

### 2.6 Gene expression stability analysis

To validate the expression stability of candidate reference genes, the results of qRT-PCR were analyzed by the geNorm[36], NormFinder[37], and BestKeeper[38] methods, respectively, according to the manufacturers’ instructions. In geNorm and NormFinder, the input data were the gene relative quantities that transferred from the raw Ct results of qRT-PCR, and the output data were the stability value of gene expression (M value). In BestKeeper, the input data were the untransformed Ct results of qRT-PCR, and the output data were a series of results that indicate the expression stability of the genes.

### 2.7 Correlation analysis between gene expressions and ginsenoside content

The relative expression levels of genes involved in ginsenoside biosynthesis normalized by the candidate reference genes and the mono-ginsenoside content in the same sample were used for the correlation analysis. The Spearman's correlation coefficient was calculated by SPSS (version 23) software to reflect the relationship between gene expressions and ginsenoside content.

## 3. Results

### 3.1. Changes of ginsenoside content in the ginseng hairy roots after the MeJA treatment

**Fig 1:**
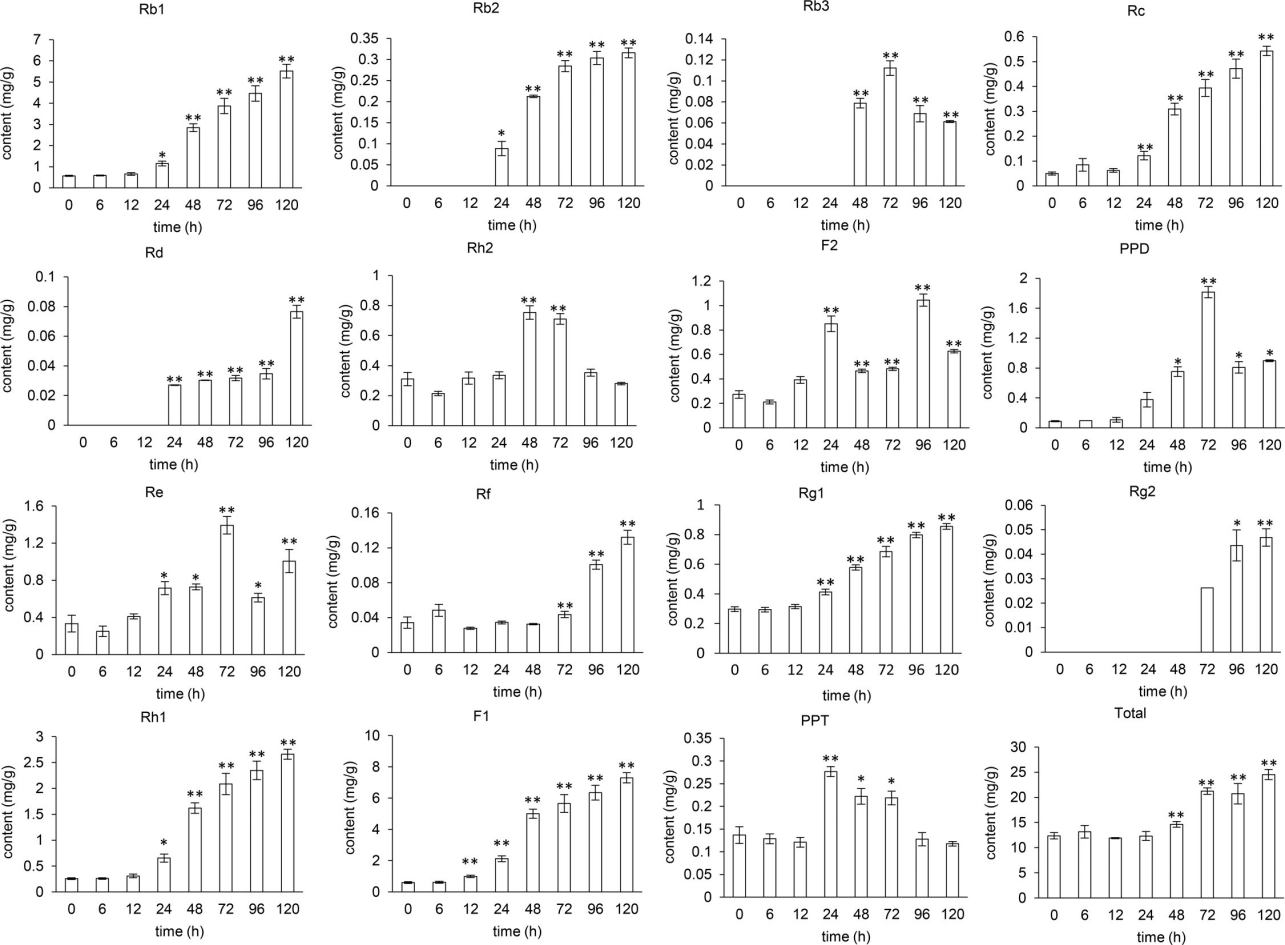
The content of ginsenosides in MeJA-treated ginseng hairy roots. The content of ginsenosides in the samples at “0” h shown in the x-axes of the figures were the average content of ginsenosides in the samples collected at 6 h to 120 h from non-treated culture after MeJA treatment. The *t*-test was used to determine the difference of ginsenoside content between the MeJA treated samples and the average content of ginsenoside in the non-treated samples. “*” for a two-tailed significance of *P* ≤ 0.05, “**” for a two-tailed significance of *P* ≤ 0.01.

The content of 14 mono-ginsenosides in the MeJA-treated hairy roots were determined. These 14 mono-ginsenosides included Rb1, Rb2, Rb3, Rc, Rd, Re, Rf, Rg1, Rg2, Rh1, Rh2, F1, F2 and CK. We also quantified the content of glycoside PPT, glycoside PPD and total ginsenoside in the MeJA-treated hairy roots. The content of mono-ginsenoside and total ginsenoside changing with MeJA-treated time were showed as Fig 1. The content of 13 mono-ginsenosides, two glycosides and total ginsenoside were significantly changed with MeJA treatment (CK have not been detected). The data of the control group (0 h) with no MeJA treatment was the average content of blank samples collected at seven-time points after MeJA treatment because the mono-ginsenoside content little changed through the 120-hour period for the untreated samples.

The changing trend of ginsenoside content showed two patterns. Nine of the 13 mono-ginsenosides and total-ginsenoside content showed constantly and significantly increasing trend, while the remaining four mono-ginsenosides and glycoside content fluctuated along with the MeJA treatment time. The constant increase of the mono-ginsenoside content indicated the accumulation of their biosynthesis while the fluctuation of the mono-ginsenoside content might reflect the dynamic status of their synthesis and decomposition as the time of MeJA treatment lasted.

The time points when the significant change of ginsenoside content initially occurred varied from 12 hours to 96 hours. The F1 mono-ginsenoside content significantly increased at 12 h after the treatment. The Re, Rg1, Rh1, Rb1, Rb2, Rc, Rd, and F2 content significantly increased at 24 h after the treatment. The Rb3, Rh2, PPD and total ginsenoside content significantly increased at 48 h after the treatment. The Rf content significantly increased at 72 h after the treatment and the Rg2 content significantly increased at 96 h after the treatment. The time points from which the significant increase of ginsenoside content in the MeJA-treated hairy roots started might suggest the positions and the order of the mono-ginsenoside in the ginsenoside biosynthesis pathway.

Moreover, we summed the content of PPT-type and PPD-type ginsenosides respectively, and analyzed their content ratio at the different time point of the MeJA treatment (Table 1). Before MeJA-treated, the content ratio of PPT-type ginsenosides to PPD-type ginsenosides in ginseng hairy roots was 1.28, but it increased along with the MeJA treatment and reached a maximum of 1.50 at 48 h after treatment. This result showed that the biosynthesis and accumulation of PPT-type ginsenosides were faster than PPD-type in the MeJA-treated ginseng hairy roots.

**Table 1:** The content ratio of PPT-type ginsenosides to PPD-type ginsenosides at different time points in MeJA-treated ginseng hairy roots.

	0 h	6 h	12 h	24 h	48 h	72 h	96 h	120 h
PPD-type ginsenoside content (mg/g)	1.292	1.195	1.537	2.954	5.449	7.701	7.547	8.319
PPT-type ginsenoside content (mg/g)	1.658	1.597	2.174	4.213	8.182	10.104	10.383	12.119
The content ratio of PPT-type to PPD-type ginsenosides	1.28	1.34	1.41	1.43	1.50	1.31	1.38	1.46

In addition, the content of F1 in ginseng hairy roots was extremely high after the MeJA treatment. It was 0.599 mg/g (0.06%) in ginseng hairy roots before the MeJA treatment and reached 7.298 mg/g (0.73%) after 120-hour MeJA treatment that was similar to the content of Rb1. This was a new finding that the F1 content could increase to the same level of Rb1 content in ginseng hairy roots under MeJA treatment. This result provides information useful for the production of ginsenoside F1, even though additional research is needed to further confirm this result.

### 3.2 Selection of reference genes desirable for qRT-PCR in the MeJA-treated ginseng hairy roots

#### 3.2.1 Expression profiles of the candidate reference genes

**Fig 2:**
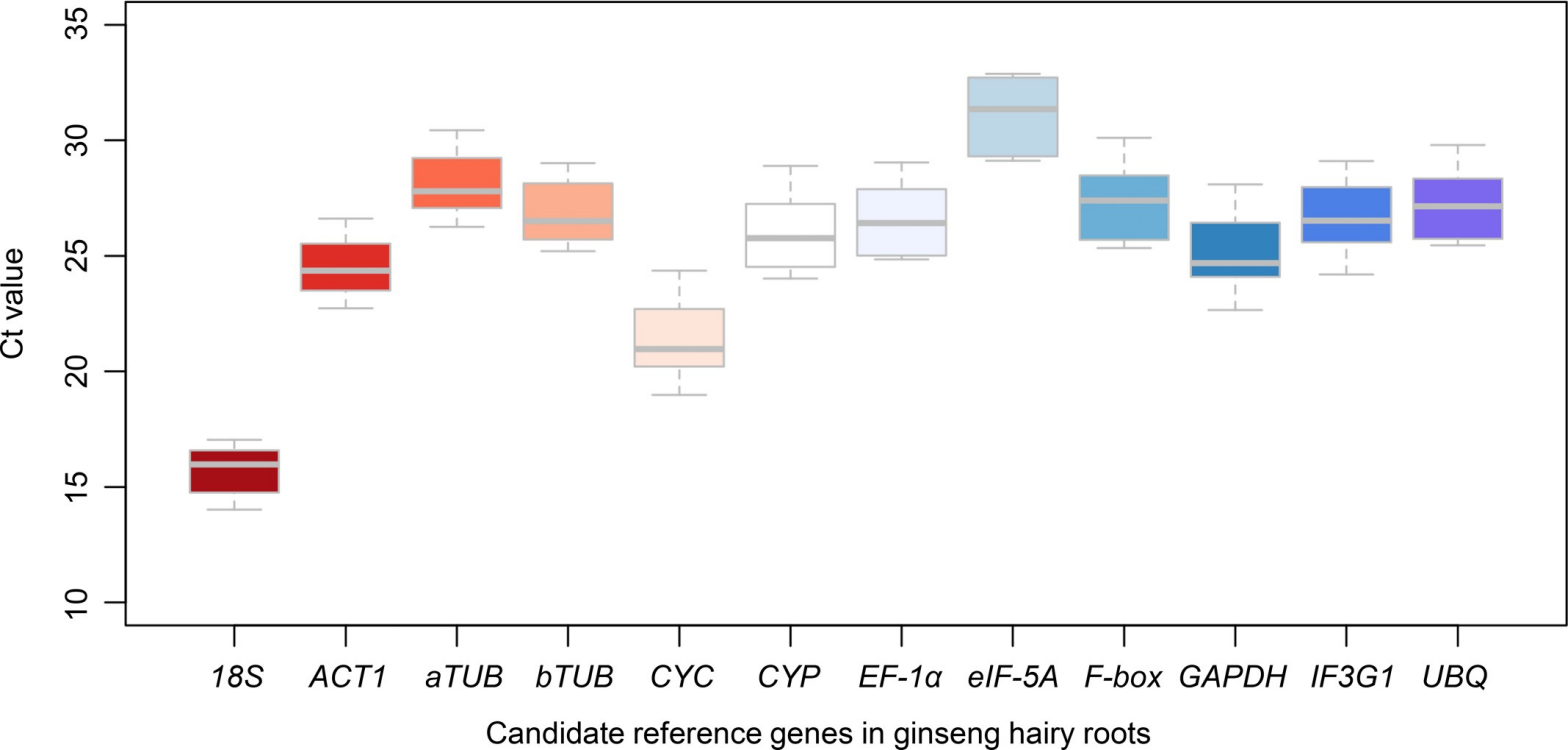
The abundance of candidate reference genes. Ct values of the candidate reference genes indicating their abundances in the samples.

The expression stabilities of all 12 candidate reference genes were preliminarily displayed by cycle threshold (Ct) values that represent the transcript abundance of the genes in the tested samples. In all samples, the Ct values of the candidate reference genes ranged from 15 to 31 (Fig 2). Among which, the *18S* (15.73±0.80) was the most abundant in the MeJA-treated hairy roots, followed by those of *CYC* (21.24±1.22), *5S* (21.98±0.67), *ACT1* (24.44±1.07), *GAPDH* (25.02±1.27), *CYP* (25.94±1.19), *EF-1α* (26.70±1.23), *IF3G1* (26.70±1.20), *bTUB* (26.73±1.19), *UBQ* (27.20±1.24), *F-box* (27.33±1.26), *aTUB* (28.02±1.17) and *eIF-5A* (31.13±1.26).

#### 3.2.2 Stability analysis by geNorm

**Fig 3:**
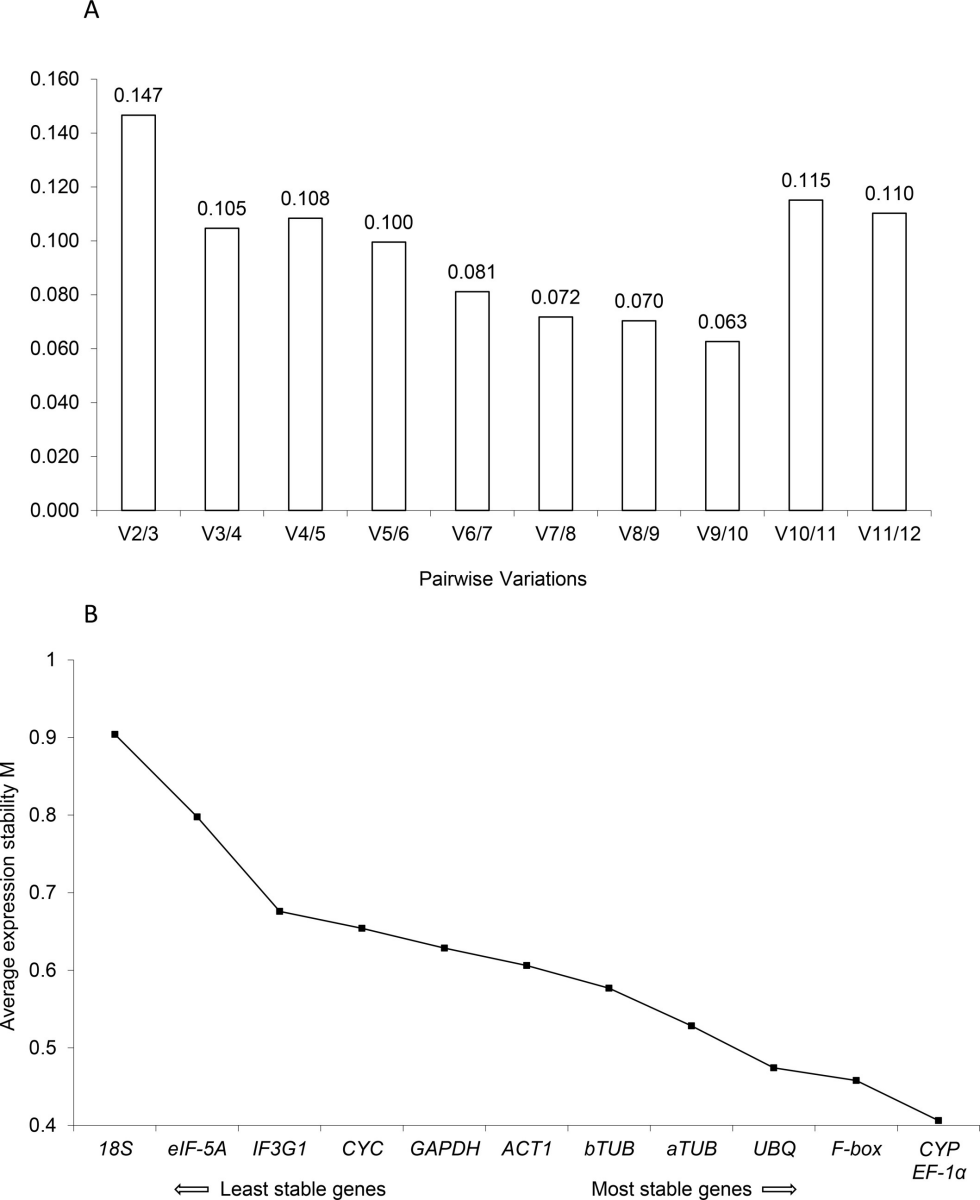
Stability analysis by geNorm. (A) The optimal numbers of control genes for normalization. (B) Average expression stability values of the candidate reference genes. The smaller M value indicates the better suitability of the gene for qRT-PCR normalization.

The pairwise variation and the M value are the two main indicators of reference gene expression stability in geNorm, which represent the suitable gene number for normalization and the most desirable reference gene. Fig 3 showed the analysis results of the 12 candidate reference genes in MeJA-treated hairy roots using geNorm. The pairwise variation values of all gene pairs were smaller than the cut-off value (0.15), suggesting that two genes should be used for qRT-PCR normalization in the hairy roots treated by MeJA. The M values of these genes in descending order were *18S*, *eIF-5A*, *IF3G1*, *CYC*, *GAPDH*, *ACT1*, *bTUB*, *aTUB*, *UBQ*, *F-box*, *CYP* and *EF-1α*. Since the most suitable reference genes for qRT-PCR normalization should have the minimum M value, the *CYP* and *EF-1α* genes were selected for qRT-PCR normalization for the hairy roots treated with MeJA.

#### 3.2.3 Stability analysis by NormFinder

The stability value based on variance analysis was the only indicator of reference gene expression stability in NormFinder. As the M value of geNorm, the smaller the stability value, the more stable the gene and the more suitable the gene for qRT-PCR normalization. The analysis results of the 12 candidate reference genes were shown in Table 2. The most stable reference gene was shown to be *CYP*, with the value of 0.234; the most unstable genes were shown to be *18S* (0.909) and *eIF-5A* (0.858), and the M values of the remaining genes were between 0.2 and 0.4. This result was in line with the results of geNorm.

**Table 2:** The expression stability of the candidate reference genes determined by NormFinder.

Gene	Stability value
*CYP*	0.234
*EF-1α*	0.255
*ACT1*	0.276
*F-box*	0.305
*GAPDH*	0.346
*UBQ*	0.360
*bTUB*	0.366
*IF3G1*	0.372
*aTUB*	0.418
*CYC*	0.432
*eIF-5A*	0.858
*18S*	0.909

#### 3.2.4 Stability analysis by BestKeeper

The standard deviation (SD), coefficient variation (CV) and correlation coefficients are the vital indicators of gene expression stability in BestKeeper. The result of the BestKeeper analysis was shown in Table 3. Only the gene with an SD value below 1.0 was desirable for qRT-PCR, so none of the 12 candidate reference genes met this criterion. Nevertheless, the correlation coefficients of all the 12 genes were quite high and the *p*-values were all < 0.001. Among the 12 candidate genes, the SD value of *18S* was the lowest (SD = 1.25).

**Table 3:** The expression stability of the candidate reference genes determined by BestKeeper.

	*18S*	*ACT1*	*aTUB*	*bTUB*	*CYC*	*CYP*	*EF-1α*	*eIF-5A*	*F-box*	*GAPDH*	*IF3G1*	*UBQ*
N	28	28	28	28	28	28	28	28	28	28	28	28
std dev [± CP]	1.25	1.63	1.88	1.86	1.96	1.93	1.96	2.06	2.00	2.01	1.93	1.98
CV [% CP]	7.92	6.68	6.72	6.98	9.22	7.45	7.39	6.63	7.30	8.04	7.21	7.27
coeff. of corr. [r]	0.87	0.98	0.97	0.98	0.97	0.99	0.99	0.89	0.99	0.99	0.98	0.98
*p*-value	0.001	0.001	0.001	0.001	0.001	0.001	0.001	0.001	0.001	0.001	0.001	0.001

### 3.3 Validation of the most desirable reference genes

#### 3.3.1 The relative expression levels of key enzyme genes involved in ginsenoside biosynthesis normalized by different reference genes

Because the ginseng hairy roots are widely used in the study of ginsenoside biosynthesis, we chose four genes encoding key enzymes in the ginsenoside biosynthesis pathway as the target genes to validate the most desirable reference genes obtained above. In the results of geNorm and NormFinder, the gene of *CYP* was the most stable reference gene and the *18S* was the most unstable gene. However, in the result of BestKeeper, the *18S* was the most stable reference gene. Therefore, the *CYP* and *18S* genes were selected as the reference genes to confirm whether they were suitable for normalization of gene expression quantified by qRT-PCR in the MeJA-treated hairy roots.

**Fig 4:**
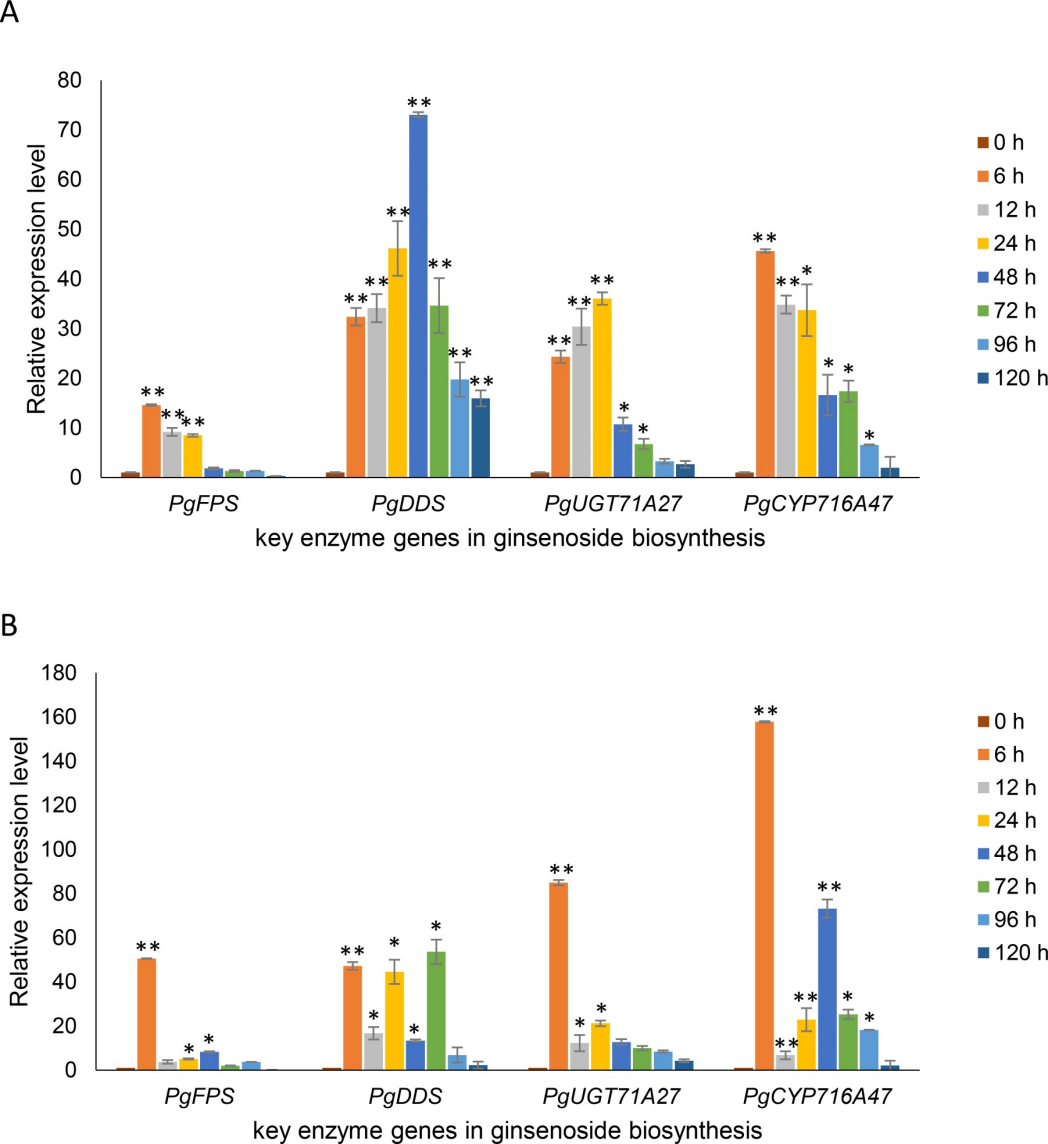
The relative expression levels of key enzyme genes involved in ginsenoside biosynthesis under MeJA treatment with *CYP* or *18S* as the reference gene. (A) Relative expression levels of the four genes correlated with ginsenoside biosynthesis with *CYP* as the reference gene. (B) Relative expression levels of the four genes correlated with ginsenoside biosynthesis with *18S* as the reference gene. The significance was calculated against the expression of no-treated hairy-roots by *t*-test. “*” for a two-tailed significance of *P* ≤ 0.05, “**” for a two-tailed significance of *P* ≤ 0.01.

The qRT-PCR was conducted for the four selected target genes with *CYP* and *18S* as reference genes. The relative expression levels of these four genes were calculated using *CYP* and *18S* as the reference genes respectively (Fig 4). When *CYP* was used as the reference gene, the relative expression levels of all four genes significantly increased compared with the no-treated hairy roots. The relative expression levels of *PgFPS* and *PgCYP716A47* reached a maximum at 6 h after the MeJA treatment and then, gradually reduced along with the time of MeJA treatment. The relative expression levels of *PgDDS* and *PgUGT71A27* reached their maximum at 48 h and 24 h, respectively, after the MeJA treatment and then gradually decreased as the time of the MeJA treatment lasted. Besides, the trends of these four genes showed good regularity. When *18S* was used as the reference gene, the relative expression levels of all four genes significantly increased only partially, and absent in regularity. Therefore, *CYP* was more desirable as the reference gene for gene expression analysis in ginseng hairy roots treated with MeJA.

#### 3.3.2 The correlation analysis of gene expression levels with ginsenoside content

To further confirm the above observation, correlation analysis was performed between the relative expressions of four key enzyme genes involved in ginsenoside biosynthesis and ginsenoside content in the MeJA-treated ginseng hairy roots (Table 4). When *CYP* was used as the reference gene, the expressions of *PgFPS* and *PgUGT71A27* were significantly correlated with the content of 11 kinds of ginsenosides; *PgDDS* and *PgCYP716A47* were significantly correlated with the content of 8 kinds of mono-ginsenosides. When *18S* was used as the reference gene, the expression of *PgFPS* was significantly correlated with the content of 8 kinds of mono-ginsenosides; *PgDDS* was significantly correlated with the content of only 1 kind of mono-ginsenoside; *PgUGT71A27* was significantly correlated with the content of 9 kinds of mono-ginsenosides; *PgCYP716A47* was not significantly correlated with the content of any kinds of mono-ginsenoside analyzed. This result further verified that *CYP* was more desirable as a reference gene than *18S* for gene expression analysis by qRT-PCR in the MeJA-treated ginseng hairy roots.

**Table 4:** Correlation analysis of key enzyme gene expressions with ginsenoside content in MeJA-treated ginseng hairy roots using *CYP* or *18S* as the reference gene.

Key enzyme genes	Reference genes		Rb1	Rb2	Rb3	Rc	Rd	Re	Rf	Rg1	Rg2	Rh1	Rh2	F1	F2	PPD	PPT	TOTAL
*PgFPS*	*CYP*	Correlation Coefficient	.872^**^	.644^*^	.497	.829^**^	.758^**^	.681^**^	.425	.875^**^	.029	.871^**^	.348	.880^**^	.632^**^	.851^**^	.009	.786^**^
		Sig. (2tailed)	.000	.013	.120	.000	.003	.003	.070	.000	.957	.000	.171	.000	.009	.000	.971	.000
	*18S*	Correlation Coefficient	.609^**^	.279	.196	.639^**^	.440	.610^**^	.261	.664^**^	.429	.589^**^	.301	.621^**^	.218	.668^**^	.053	.516^*^
		Sig. (2tailed)	.004	.334	.564	.002	.133	.009	.280	.004	.397	.006	.240	.003	.418	.002	.836	.024
*PgDDS*	*CYP*	Correlation Coefficient	.519^*^	.723^**^	.241	.532^*^	.538	.137	.647^**^	.653^*^	.314	.507^*^	.348	.483^*^	.297	.139	.366	.579^**^
		Sig. (2tailed)	.019	.003	.474	.016	.058	.599	.003	.011	.544	.023	.171	.031	.264	.581	.135	.009
	*18S*	Correlation Coefficient	.329	.490	.565	.377	.467	.005	.370	.468	.486	.295	.395	.337	.279	.117	.610^**^	.363
		Sig. (2tailed)	.156	.075	.070	.101	.108	.985	.119	.091	.329	.207	.117	.146	.295	.645	.007	.126
*PgUGT71A27*	*CYP*	Correlation Coefficient	.859^**^	.903^**^	.109	.808^**^	.863^**^	.559^*^	.602^**^	.775^**^	.314	.860^**^	.233	.833^**^	.459	.743^**^	.154	.875^**^^**^
		Sig. (2tailed)	.000	.000	.749	.000	.000	.020	.006	.000	.544	.000	.368	.000	.074	.000	.542	.000
	*18S*	Correlation Coefficient	.648^**^	.556^*^	.210	.701^**^	.714^**^	.419	.358	.645^**^	.314	.627^**^	.213	.660^**^	.371	.646^**^	.170	.658^**^
		Sig. (2tailed)	.002	.039	.536	.001	.006	.094	.132	.005	.544	.003	.411	.002	.158	.004	.499	.002
*PgCYP716A47*	*CYP*	Correlation Coefficient	.516^*^	.464	.647^*^	.474^*^	.505	.213	.033	.051^*^	.657	.514^*^	.328	.574^**^	.450	.494^*^	.121	.461^*^
		Sig. (2tailed)	.020	.095	.031	.035	.078	.411	.892	.037	.156	.020	.198	.008	.080	.037	.633	.047
	*18S*	Correlation Coefficient	.141	.015	.433	.180	.099	.130	.139	.284	.086	.110	.135	.214	.009	.117	.263	.095
		Sig. (2tailed)	.552	.958	.184	.446	.748	.619	.571	.269	.872	.645	.606	.366	.974	.645	.291	.700

“*” for a two-tailed significance of *P* ≤ 0.05

“**” for a two-tailed significance of *P* ≤ 0.01

## 4. Discussion

### 4.1 The changes of different type mono-ginsenoside content under MeJA treatment

Methyl jasmonate as the elicitor of secondary metabolism can induce the accumulation of ginsenosides in ginseng. Kim et al.[39] reported that the ginsenosides of the Rb group accumulated more than that of the Rg group under the MeJA treatment. Kang et al. [40] and Oh et al.[18] reported that MeJA treatment could significantly enhance only PPD-type ginsenosides content. This study showed that the content of all the detected mono-ginsenosides and glycosides increased in hairy roots after the MeJA treatment. Moreover, we showed that the accumulation of the PPT-type ginsenosides was faster than that of the PPD-type ginsenosides under the MeJA treatment, which was different from the results reported before.

There may be two reasons that led to the difference among these studies. The one may be due to the different plant materials used for the studies and the other could be attributed to different mono-ginsenosides detected. The types and content of secondary metabolites as well as synthetic substrates and enzymes[41] may differ in different plant materials. Kim et al.[39] and Kang et al.[40] used the adventitious roots of ginseng for their studies, and Oh et al.[18] used fine root, root body, epidermis, rhizome, stems and leaf for their studies while we used hairy roots as materials in this study. However, we consider the types of detected mono-ginsenosides was the major reason for the observed discrepancy. The numbers of PPD-type mono-ginsenosides and PPT-type mono-ginsenosides detected by Oh et al.[18] were four and four, respectively. Those detected by Kang et al.[40] were sixteen and four, while ours were eight and seven. As more than 150 naturally occurring ginsenosides have been identified from *Panax* species, even if some of them account for a large proportion, we still cannot conclude that the MeJA treatment influences only certain types of ginsenosides. Moreover, the mechanism of MeJA in secondary metabolism is complicated. Several factors have been identified to play a vital role in the induction of ginsenoside biosynthesis, including JASMONATE ZIM DOMAIN (JAZ) proteins[42], transcription factors (TFs)[43], phytohormones[44] and some enzyme-coding genes[45]. All these factors were shown to have a global influence on secondary metabolisms, except for enzyme-coding genes. However, enzyme-coding genes may also have multiple functions that a single enzyme may catalyze biosynthesis of multiple secondary metabolites[8]. Hence, the influence of MeJA on secondary metabolism is likely to be overall, instead of metabolite-specific. Therefore, we should consider the induction of ginsenosides by MeJA from a global perspective.

### 4.2 The validation of *CYP* as the reference gene in ginseng hairy roots under MeJA treatment

By using gene expression quantified with qRT-PCR, followed by correlation analysis with the ginsenoside content variation, the genes involved in ginsenoside biosynthesis can be confirmed, at least preliminarily. Therefore, the stably expressed reference genes used for qRT-PCR have become a significant factor of properly quantifying gene expressions. In fact, several reference genes have previously been used in ginseng. The *β-actin* gene has been the most commonly used reference gene for all types of ginseng materials, including hairy roots[46], adventitious roots[47], different tissues[48], and transgenic materials[49]. However, it did not perform well in this study. Liu et al.[21] reported that the *CYP* and *EF-1α* genes were the most stable reference genes at different growth stages and in different organs of ginseng. Wang et al.[22] found that the *EF1-γ*/*IF3G1* genes were the most stable reference genes in different tissues and that the *IF3G1*/*ACT11* were the most stable reference genes in seedlings grown under heat stress condition. In this study, we investigated the stability of 12 candidate reference genes including 10 traditional housekeeping genes tested by Liu et al.[21] and two other housekeeping genes tested by Wang et al.[22]. Our results showed that the *CYP* gene was the most stable reference gene that was consistent with the result of Liu et al.[21].

To further confirm whether *CYP* is the most suitable reference gene in ginseng hairy roots under the MeJA treatment, four genes that encode key enzymes involved in ginsenoside biosynthesis were used as the positive controls. The expression of *PgFPS* was up-regulated under MeJA treatment as reported by Kim et al.[32]. *PgCYP716A47* also responded to MeJA treatment as reported by Han et al.[35]. *PgUGT71A27*[34] and *PgDDS*[33] were reported to participate in ginsenoside biosynthesis. Since they have been already showed to relate with ginsenoside biosynthesis, it is expected that their expressions should be also correlated with the change of ginsenoside content. Our results showed that the relative expression levels of the genes used *CYP* as the reference gene were more significantly correlated with the ginsenoside content than those used *18S* as the reference gene. Especially for *PgCYP716A47*, its expression was significantly correlated with the content of 8 kinds of ginsenosides when *CYP* was used as the reference gene, while its expression had no significant relevance with the content of any kinds of ginsenosides when *18S* was used as the reference gene. These results indicate that our analysis of the candidate reference genes is reliable and the *CYP* gene can be used as the reference gene for qRT-PCR when studying the genes involved in ginsenoside biosynthesis. This study lays a foundation for further explorations of the genes involved in ginsenoside biosynthesis and the molecular mechanism underlying the process.

## Supporting information

S1 Fig The induction and culture of ginseng hairy roots. (A) Ginseng sterile seedlings. (B) and (C) Putative ginseng hairy root inducing from the explants. (D) and (E) The culture of single ginseng hairy root. (F) The ginseng hairy roots cultured by liquid medium for 23 days.
(TIF)

S2 Fig The chromatogram of standard products and samples. (A) The chromatogram of single standard products. (B) The chromatogram of multiple standard products. (C) The chromatogram of samples. The type and retention time of mono-ginsenoside were showed within every single chromatographic peak.
(TIF)

## Abbreviations

MeJA: Methyl jasmonate
qRT-PCR: Quantitative real-time polymerase chain reaction
PPD: Protopanaxadiol-type ginsenoside
PPT: Protopanaxatriol-type ginsenoside
HPLC: High-performance Liquid Chromatography
SD: Standard Deviation
CV: Coefficient of Variation
