# Supplementary figures and images for: Selection and validation of reference genes desirable for gene expression analysis by qRT-PCR in MeJA-treated ginseng hairy roots
Source: PLoS One. 2019 Dec 5;14(12):e0226168. doi: 10.1371/journal.pone.0226168 (PMC6894815; doi:10.1371/journal.pone.0226168)

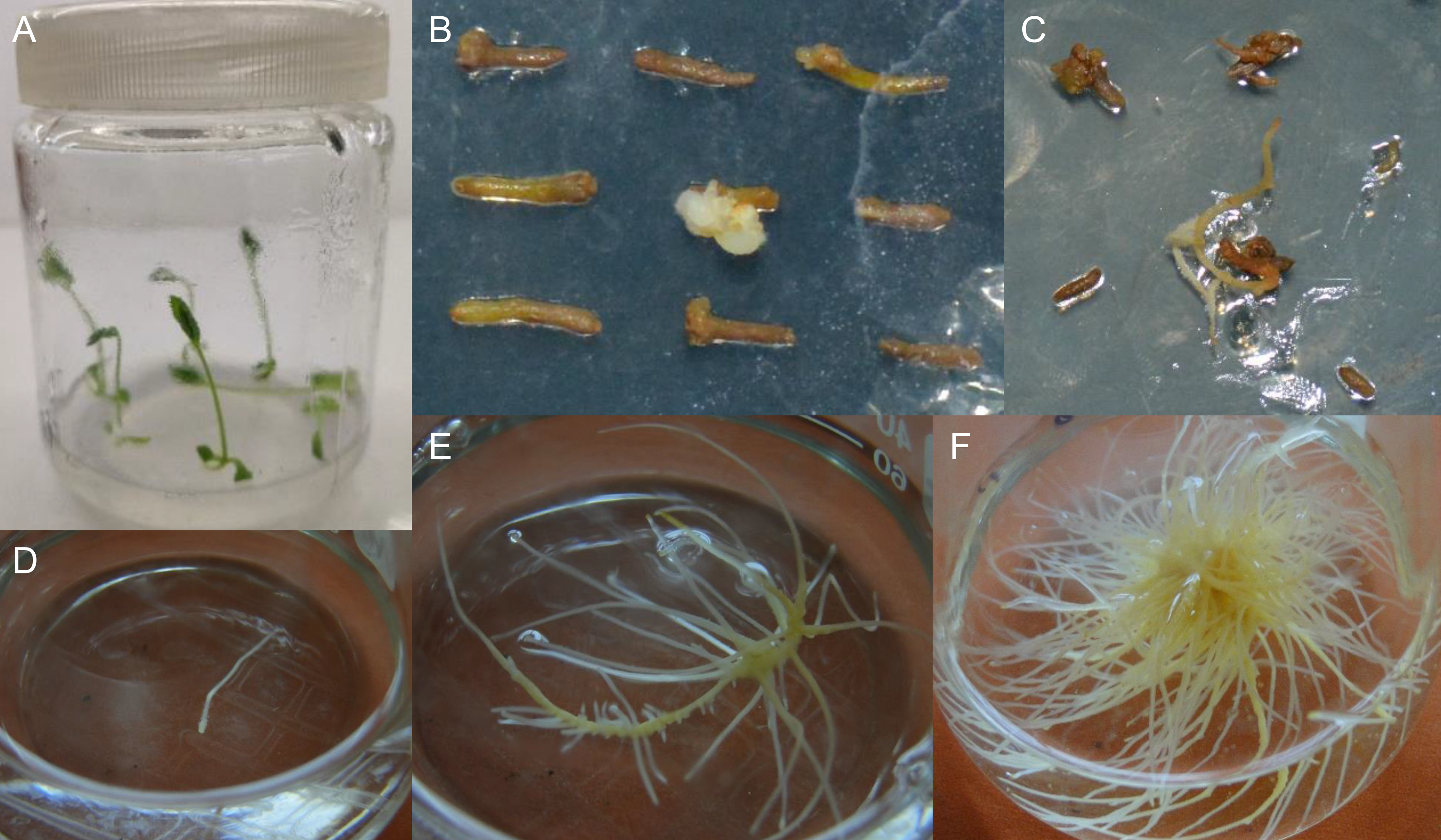

Supplement: S1 Fig — (A) Ginseng sterile seedlings. (B) and (C) Putative ginseng hairy root inducing from the explants. (D) and (E) The culture of single ginseng hairy root. (F) The ginseng hairy roots cultured by liquid medium for 23 days. (TIF) [file pone.0226168.s001.tif]

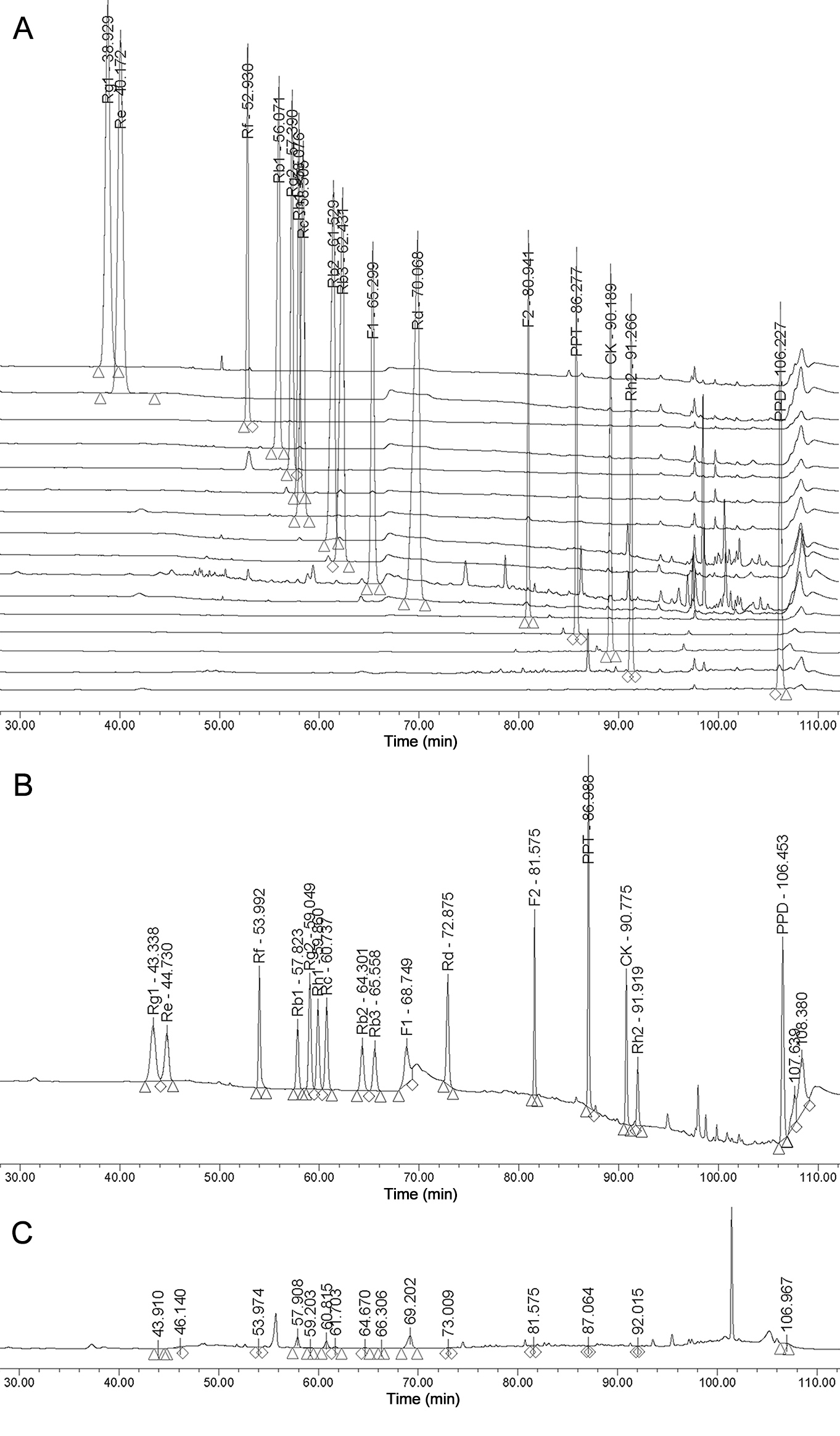

Supplement: S2 Fig — (A) The chromatogram of single standard products. (B) The chromatogram of multiple standard products. (C) The chromatogram of samples. The type and retention time of mono-ginsenoside were showed within every single chromatographic peak. (TIF) [file pone.0226168.s002.tif]
